# Observations of the Morning Development of the Urban Boundary Layer Over London, UK, Taken During the ACTUAL Project

**DOI:** 10.1007/s10546-017-0300-z

**Published:** 2017-10-29

**Authors:** Christos H. Halios, Janet F. Barlow

**Affiliations:** Department of Meteorology, University of Reading, Earley Gate, PO Box 243, Reading, RG6 6BB UK

**Keywords:** Low-level jets, Morning transition, Turbulent kinetic energy budget, Urban boundary layer, Urban mixing height

## Abstract

The study of the boundary layer can be most difficult when it is in transition and forced by a complex surface, such as an urban area. Here, a novel combination of ground-based remote sensing and in situ instrumentation in central London, UK, is deployed, aiming to capture the full evolution of the urban boundary layer (UBL) from night-time until the fully-developed convective phase. In contrast with the night-time stable boundary layer observed over rural areas, the night-time UBL is weakly convective. Therefore, a new approach for the detection of the morning-transition and rapid-growth phases is introduced, based on the sharp, quasi-linear increase of the mixing height. The urban morning-transition phase varied in duration between 0.5 and 4 h and the growth rate of the mixing layer during the rapid-growth phase had a strong positive relationship with the convective velocity scale, and a weaker, negative relationship with wind speed. Wind shear was found to be higher during the night-time and morning-transition phases than the rapid-growth phase and the shear production of turbulent kinetic energy near the mixing-layer top was around six times larger than surface shear production in summer, and around 1.5 times larger in winter. In summer under low winds, low-level jets dominated the UBL, and shear production was greater than buoyant production during the night-time and the morning-transition phase near the mixing-layer top. Within the rapid-growth phase, buoyant production dominated at the surface, but shear production dominated in the upper half of the UBL. These results imply that regional flows such as low-level jets play an important role alongside surface forcing in determining UBL structure and growth.

## Introduction

The diurnal cycle of the atmospheric boundary layer (ABL) due to surface heating exerts strong control on the surface climate and scalar concentrations. The typical diurnal evolution of the ABL over land has been described previously (e.g. Stull 1988; Garratt 1992) as the succession of four basic phases: (1) Formation of a shallow convective boundary layer (CBL) near the ground; (2) Rapid growth of the CBL, up to the level of the capping inversion; (3) Consolidation of the well-mixed CBL where the mixing height increases only slowly and is influenced by large-scale vertical motions at the CBL top, and (4) Decay of thermally-driven turbulence and vertical mixing followed by the formation of a shallow stable layer close to the ground. This typical evolution is most clearly evident during clear, sunny days.

Examination of the urban boundary layer (UBL) during transition periods has important implications for air quality, since the timing of high emissions occurs mainly during morning and evening traffic rush hours. For example, in a recent study that reports on more than three years of measurements of fluxes of methane (CH$_{4}$), carbon monoxide (CO) and carbon dioxide (CO$_{2}$) from eddy-covariance measurements in central London, UK (Helfter et al. 2016) it was found that emissions increased sharply from 0600 LST when traffic rates increase, and reached a daytime maximum at around 1200 LST. A time lag on the order of 1 h was observed in the measured fluxes between a height of 190 m and a lower height of 50 m, mainly in winter, and this was attributed to reduced turbulent mixing. However, modelling the boundary layer can be most difficult when it is in transition—passing from night to day, or day to night—and when forced by a complex surface, such as an urban area. In particular, from a pollution perspective, the mixing height (*MH*) is of great importance. Mixing height is defined as the height of the layer adjacent to the ground over which pollutants or any constituents emitted within this layer or entrained into it become vertically dispersed by convection or mechanical turbulence within a time scale of about an hour (Seibert et al. 2000). Therefore it determines the volume available for pollutant dispersion through the action of turbulence. In the present study the mixing height is identified as the depth of the turbulence layer adjacent to the ground.

The vast majority of studies tackling morning boundary-layer development have focused on the rapid-growth phase. Since the late 1960s numerous observational (e.g. Kaimal et al. 1976; Batchvarova and Gryning 1991) and theoretical (e.g. Pino et al. 2003; Conzemius and Fedorovich 2006; Sorbjan 2007) studies have focused on different aspects of morning development and the physical processes that are involved in boundary-layer growth are well established. Thus the boundary layer begins to grow due to increasing surface heat flux, and also entrainment, when more buoyant air from the free atmosphere is engulfed by convective thermals and becomes part of the boundary layer (Conzemius and Fedorovich 2006). These processes can be significantly modified by the presence of wind shear at the surface and across the entrainment layer (Fedorovich et al. 2001).

Until recently, little attention had been given to the first stage of boundary-layer morning development that consists of a shallow CBL that grows slowly until the nocturnal inversion has been completely eroded, possibly with no significant interfacial mixing (Carson 1973; Sorbjan 2004). Stull (1988) identified a stable boundary layer (SBL) capping a shallow CBL with a depth on the order of tens to hundreds of metres. In one of the first studies to specifically focus on the so-called morning transition, Angevine et al. (2001) defined the beginning of this phase as the time when the surface sensible heat fluxes change sign (“cross-over” in sensible heat flux). The end of this phase was defined as when the mixing height first reaches 200 m: this height was selected on practical grounds (i.e. the sensors on the meteorological tower were 200 m above ground level (a.g.l.)). It was also reported that the duration of the morning transition was reduced under enhanced wind speeds before and during the morning-transition phase. The role of wind shear was also investigated by Lapworth (2006), based on six years of observations.

The evolution from a nocturnal SBL through the morning transition to a fully developed CBL was studied by Beare (2008) using large-eddy simulation (LES). The morning transition was found to have features of both the CBL and SBL, and therefore it was named the “mixed CBL-SBL” phase. It was shown that interfacial mixing did occur at the top of the boundary layer during the morning transition, but entrainment depended on surface friction velocity as well as convection, and the ratio of entrainment to surface heat flux was much larger than for a fully-developed CBL, as observed by Angevine et al. (2001).

CBL evolution has been studied for homogeneous, equilibrium and stationary flows over relatively smooth, homogeneous surfaces. The large spatial heterogeneity in roughness, heat and moisture of an urban surface determines surface energy budget and flow characteristics. Hence, the whole boundary layer, its stability, thermodynamic properties, and the mixing height are affected (Christen and Vogt 2004; Barlow 2014). In particular: large roughness elements lead to more sheared, heterogeneous flows (Barlow 2014); greater partitioning into sensible heat flux due to a lack of moisture may lead to a deeper boundary layer (Banta and White 2003); urban heat islands induce urban convergence zones that may be related to convective thunderstorm initiation on the lee-side of the city (Bornstein and Lin 2000); the heat storage in urban surfaces leads to delayed warming/cooling (Kotthaus and Grimmond 2014), and therefore nocturnal stable conditions generally develop later (Barlow et al. 2015).

Roth (2000) reviewed urban turbulence observations, and noted a distinct lack of measurements throughout the depth of the UBL, due to practical challenges and flow complexity. Thus, most studies relating to boundary-layer development are either observational and conducted in homogeneous rural environments (e.g. Angevine et al. 2001; Garcia et al. 2002; Lapworth 2006), or idealized modelling studies (e.g. Pino et al. 2003; Conzemius and Fedorovich 2006; Sorbjan 2007; Beare 2008). Some studies have examined the seasonal variation of the rural CBL (Schween et al. 2014), but little is known about this variation over the urban surface, e.g., Angevine et al. (2003) found that the urban mixing height could be up to 700 m higher than the rural mixing height during the summer. Barlow et al. (2015) observed “upside down” turbulence characteristics in the morning UBL associated with shear due to persistent nocturnal low-level jets (LLJs) above. Other studies of heterogeneous boundary layers include Lenschow et al. (1979), who showed that complex orography can shorten the duration of the temperature transition. Wildmann et al. (2015) used airborne turbulence measurements to study the morning transition over heterogeneous terrain and found that mixed-layer scaling is appropriate for some parameters.

The aim of the present study is thus to test whether the evolution of the UBL from night time until its fully-developed convective state is distinctly different to the evolution of a rural boundary layer, using a novel combination of ground-based remote sensing and in situ instrumentation. Particular aims include:

1. Determining (a) the duration of the morning-transition phase, and (b) the growth rate of the UBL, and testing their dependence on various. physical variables.
2. Examining the importance of regional atmospheric flows (such as LLJs) versus surface-driven forcing.
3. Quantifying wind shear throughout the depth of the UBL during its evolution, and,
4. Testing whether shear or buoyant processes dominate at different heights throughout the UBL evolution.

The seasonal dependence of the results is also examined.

## Methods

### Experimental Sites and Instrumentation

Remote and in situ instrumentation platforms were deployed in London for the ACTUAL project (Advanced Climate Technology Urban Atmospheric Laboratory), which investigated the UBL with applications to wind engineering (Barlow et al. 2011a, 2015; Lane et al. 2013; Wood et al. 2013; Drew et al. 2013). Measurements were also used for the ClearfLo (Clean Air for London) project (Bohnenstengel et al. 2015) with applications to air quality. A heterodyne scanning Doppler lidar (Stream Line, Halo Photonics) was used for observing the UBL structure (Wavelength: 1.5 $\upmu$m, Pulse repetition frequency: 10 kHz, Sampling Frequency: 50 MHz). Integrated signals were output every 3.6 s (sampling rate of 0.286 Hz), and the return signal was resolved into 30-m range gates. Returns were spurious in the first three range gates due to the geometry of the transmitter and receiver, therefore results are presented from the fourth range gate upwards (mid-point 105 m above the lidar).

**Fig. 1:**
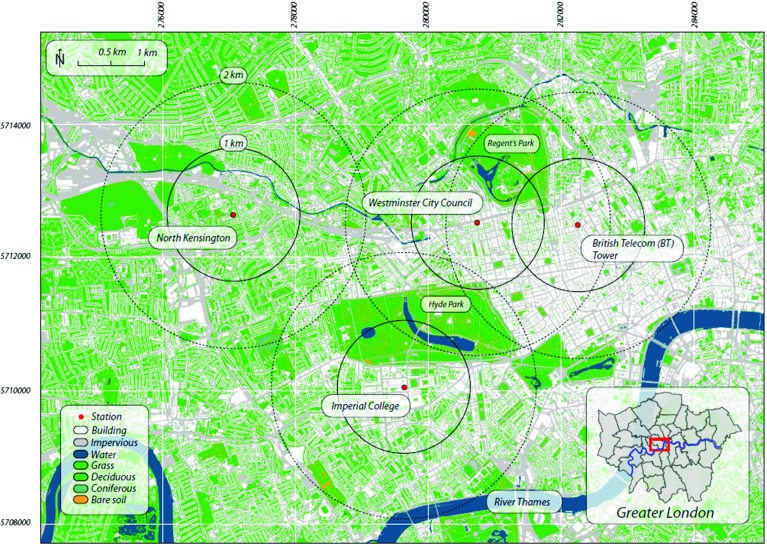
Land cover map (based on OS MasterMap) of Central London with locations where instrumentation was placed: North Kensington, Imperial College, British Telecom (BT) Tower and Westminster City Council (WCC). Map of the Greater London Area is shown in the right inset. Rings are radii of 1 km from measurement locations. The grid is labelled as metres (easting, northing), UTM zone 31N

The location of the instruments is shown in Fig. 1. For the period from 19 May 2011 to 11 January 2012 the lidar was installed on the roof-top of Westminster City Council’s building (WCC: 15 m a.g.l., 3 m above roof-top, latitude $51{^{\circ }}31^{\prime }17^{\prime \prime }\hbox {N}$, longitude $0{^{\circ }} 09^{\prime } 38^{\prime \prime }\hbox {W}$). Then the instrument was moved to the Engineering Building at Imperial College (IC: 33 m a.g.l., latitude $51{^{\circ }}29^{\prime }55^{\prime \prime }\hbox {N}$, longitude $0{^{\circ }}10^{\prime }29^{\prime \prime }\hbox {W}$) until 8 February 2012. For the period 23 July 2012 to 17 August 2012 an identical lidar was operating at North Kensington as part of the ClearfLo project (NK: ground level, latitude $51{^{\circ }}31^{\prime }15^{\prime \prime }\hbox {N}$, longitude $0{^{\circ }} 12^{\prime } 49^{\prime \prime }\hbox {W}$) with a gate resolution of 18 m. Data used herein are drawn from all three sites, and thus intra-urban differences are neglected: separation between sites is between 2.7 and 3.7 km.

The lidars were operated in two modes: vertical stare, and Doppler beam swinging (DBS). In DBS mode the lidar beam was tilted in three orthogonal directions: zenith, and 15${^{\circ }}$ off-zenith to the north and east. The DBS scan lasted approximately 21 s, and occurred at 120-s intervals, with the lidar being in vertical stare mode for the intervening 99 s, sampling every 3.6 s. In DBS mode, 30 wind-speed profiles were obtained every hour from which the mean profile was calculated. The hourly mean attenuated backscatter and vertical profiles were calculated from vertical stare mode data. Linear regression of hourly mean wind speeds calculated from DBS scans against a sonic anemometer on the BT Tower gave a slope of 0.99 (Lane et al. 2013).

Two identical turbulent flux instrumentation platforms were placed at the WCC site (3 m above roof-top), and on the British Telecom (BT) Tower (191 m a.g.l., latitude $51{^{\circ }}31^{\prime }17^{\prime \prime }\hbox {W}$, longitude $0{^{\circ }}08^{\prime }20^{\prime \prime }\hbox {N}$). Each platform had an eddy-covariance system, a weather station (Vaisala WXT520) and a net radiometer (Kipp and Zonen CNR4). The eddy-covariance system consisted of an ultra-sonic anemometer (R3-50, Gill Instruments Ltd) and infra-red gas analyzer (LI-COR 7500) and data were sampled at 20 Hz. Data from the weather station and net radiometer were sampled at 1 and 0.05 Hz respectively. At the BT Tower, the sonic anemometer was placed on a mast on the top of an open lattice scaffolding tower of height 12.3 m on top of the building to avoid flow distortion (Barlow et al. 2011). Hourly means of turbulence variables were calculated and full processing details can be found in Wood et al. (2010) and Barlow et al. (2015). Due to practical difficulties in releasing balloons or running instruments such as RASS (Radio Acoustic Sounding System) added to sodar in London, temperature profiles were not measured.

### Site Characterisation

The scalar concentration source area for the WCC site was estimated from wind-tunnel experiments to lie within a 250-m radius (Brocklehurst 2015). The mean building height within a 250-m radius was $$\bar{h}=21.4$$ m with standard deviation $$\sigma _h =11$$ m (Barlow et al. 2009), meaning that the sensor height ($$z_{m})$$ was low relative to the urban canopy, $$z_m =0.9 \bar{h} $$. It was not permitted to raise the instrumentation to a higher height at this site, i.e. to 2–3$$\bar{h}$$, estimated to be within the inertial sub-layer. Around the WCC site, the buildings were densely packed (plan area ratio of buildings $$\lambda _p =0.50$$, frontal aspect ratio $$\lambda _f =0.19)$$. The vegetation fraction within the source area was $$\lambda _v \approx 0.1$$, consisting mainly of deciduous trees planted within streets or small park areas (Lane 2014). The scalar flux source area under unstable conditions for the BT Tower was estimated to lie within a 2 and 22 km radius of the site (Helfter et al. 2011). The mean building height within 10 km of the Tower was $$8.8 \pm 3$$ m (Wood et al. 2010), meaning that for the BT Tower measurement $$z_m =22 \bar{h} $$.

Given that the WCC observations are relatively low, they are likely to be in the roughness sub-layer and therefore underestimate the inertial sub-layer fluxes: an attempt to quantify this underestimation follows. Using experimental data from the BUBBLE campaign (Basel Urban Boundary Layer Experiment), Christen (2005) derived a relationship for sensible heat flux *H* at height *z* within the roughness sub-layer as a function of heat flux in the inertial sublayer $H_{IS}$,

1 $$\begin{aligned} H\left( z \right) =H_{IS} e^{-k} \quad \hbox {for}\,z\le z_e . \end{aligned}$$

where

2 $$\begin{aligned} k=c_h \frac{\left( {z-z_e } \right) }{z_e }, \end{aligned}$$

and $z_e$ is the effective building height, defined by the inflection point in the spatially-averaged wind profile. For the non-uniform canopy found at the BUBBLE site, $z_e =1.2 \bar{h}$ and $c_h$ is an empirically-derived coefficient ($c_h =1.4$). The BUBBLE site morphological parameters are $\bar{h} =14.6\,\hbox {m}$ with standard deviation $\sigma _h =6.9\hbox {m}$, $\lambda _p =0.54$, $\lambda _f =0.37$, and $\lambda _v =0.16$.

**Table 1:** Characteristics of low, medium and high wind-speed classes for each period of UBL evolution in summer and winter

Class	Days (Lidar location)		Number of observations (h)	Mean wind speed at BT ($\hbox {m}\,\hbox {s}^{-1}$)		
				Night-time $U_{NT}$	Morning transition $U_{MT}$	Rapid growth $U_{RG}$
Summer						
S1	1 June 2011	WCC	191	4	2.7	2.6
	10 June 2011	WCC		5.3	3.2	3.2
	30 June 2011	WCC		3.3	4.8	2.6
	3 July 2011	WCC		7.7	6.5	2.4
	4 July 2011	WCC		3.8	1.7	1.7
	5 July 2011	WCC		4.5	4.5	2.3
	11 July 2011	WCC		3.9	3.6	2.5
	15 July 2011	WCC		4.3	1.7	2.7
	25 July 2011	WCC		5.9	–*	4.1
	22 August 2011	WCC		4.5	4.1	2.8
S2	24 June 2011	WCC	147	6.3	6.5	4.3
	27 June 2011	WCC		7.9	8.1	5.3
	29 June 2011	WCC		6.9	5.5	5.5
	1 July 2011	WCC		8	9.1	4.5
	10 August 2011	WCC		1.1	2.2	7.5
	15 August 2011	WCC		5	4.2	4.2
	29 August 2011	WCC		7.6	6.5	6.3
	11 August 2012	NK		5.6	–**	–**
	12 August 2012	NK		8.7	7.1	6.5
S3	3 June 2011	WCC	32	8.3		7.2
	12 July 2011	WCC		7.2	7.6	8.3
Winter						
W1	14 January 2012	IC	70	4.6	3.3	2.3
	15 January 2012	IC		8.1	7.3	6.5
	16 January 2012	IC		7.3	7.7	5.5
W2	13 January 2012	IC	70	9.8	7.5	5.0
	23 January 2012	IC		9.3	9.1	6.6
	27 January 2012	IC		9.7	8.4	6.1
W3	12 December 2011	WCC	48	11.4	10.4	9.9
	22 December 2011	WCC		9.3	11.6	10

Sites are Westminster City Council (WCC), North Kensington (NK) and Imperial College (IC)

* No points fall within the morning-transition period determined with the method described in Sect. 3.2

** There were insufficient data to define the end of the morning-transition period

Given that the values of $\sigma _h/\bar{h}$ and $\lambda _p$ for the WCC and BUBBLE sites agree to within 10%, it is assumed that the BUBBLE coefficients can be used for the WCC site. Differences in $\lambda _f$ and $\lambda _v$ are larger, and differences in site material properties will also influence heat exchange, but in the absence of a theoretical framework to understand height variations of heat flux within urban canopies, Eq. 1 has been applied to the WCC and BT Tower observations. If it is assumed that the BT Tower lies within the inertial sub-layer during the day when the mixing height is large, such that $H_{IS} \approx H_{BT}$, then Eq. 1 gives $H_{WCC}/H_{BT} \approx 0.67$. During the rapid-growth period when both WCC and BT Tower observations lie within the CBL, the observed median of $H_{WCC}/H_{BT}$ is 0.78 for the dataset used. Differences in the source area urban fraction for the BT Tower and WCC might contribute further to the discrepancy but this topic is beyond the scope of our study. This analysis suggests that $H_{WCC}$ does underestimate the inertial sub-layer heat flux, but in an understandable way, and will be referred to in Sect. 3.1.1 when interpreting sensible heat-flux behaviour.

In this paper the impact of intra-urban variability on UBL properties is not considered and observations from all sites are combined. Two lidars were operated simultaneously at the WCC and NK sites (Fig. 1) for the period 28 July to 17 August 2012. A good agreement was found between the mixing height at the two sites ($\textit{MH}_{WCC} = 0.87\textit{MH}_{\textit{NK}} +\mathrm{263\,m}$, $R = 0.87$), justifying this approach, but intra-urban variability will be addressed in a future study.

### Data Categorisation and Composite Analysis

Given that wind speed and shear influence boundary-layer evolution, data were categorized into low, medium and high wind speed classes for summer and winter. Only hours when data from all sites and instruments were available were included. Days were categorized according to BT Tower wind speed during a representative period. Since the sunrise time changes significantly at this mid-latitude site (from 0443 UTC in June to 0806 UTC in December), the averaging period 0300 to 1400 UTC was selected because it includes all boundary-layer phases throughout the year. The values 4.5, 7.15 and 9.35 m $\hbox {s}^{-1}$ correspond to the 25th, 50th and 75th percentiles of the distribution of the wind speed at the BT Tower averaged between 0300 and 1400 UTC ($\bar{U} _{BT0314}$) and were used to define low, medium and high wind speed classes for summer and winter. For summer (June, July, August) the wind speed classes were:

S1: $$\bar{U} _{BT0314} <4.5\,\hbox {m\,s}^{-1}$$,
S2: $$4.5\,\hbox {m\,s}^{-1}<\bar{U}_{BT0314} <7.15\,\hbox {m\,s}^{-1}$$,
S3: $$7.15\,\hbox {m\,s}^{-1}<\bar{U}_{BT0314} <9.35\,\hbox {m\,s}^{-1}$$.

For winter (December, January, February), as the wind speed was generally higher, the wind-speed classes were:

W1: $$\bar{U}_{BT0314} <7.15\,\hbox {m\,s}^{-1},$$
W2: $$7.15\,\hbox {m\,s}^{-1}<\bar{U}_{BT0314} <9.35\,\hbox {m\,s}^{-1},$$
W3: $$\bar{U}_{BT0314} >9.35\,\hbox {m\,s}^{-1}.$$

Further details of the wind-speed classes can be found in Table 1, including the mean wind-speed in each boundary-layer phase.

Composite plots were constructed as follows: a new timeline with respect to sunrise was defined for each day, and then data for each variable for all days within each wind speed class were sorted and ranked based on the new timeline. A composite average was calculated by block averaging available points for each hour (between 2 and 7) in the ranked time series. Variability around the composite average is represented in the figures by the maximum and minimum values during each hour.

### Data Analysis

The lidar measurements of vertical velocity variance profiles, $\sigma _w^2 \left( z \right)$, in stare mode were corrected to allow for the relatively low sampling rate (0.287 Hz), according to the spectral correction method presented in Barlow et al. (2015). Hourly mixing heights (*MH*) were calculated from $\sigma _w^2 \left( z \right)$ as the height up to which $\sigma _w^2 >0.1\,\hbox {m}^{2}\,\hbox {s}^{-2}$ (Barlow et al. 2015). For hours when the mixing height was below the lowest observable height, *MH* was set to the mid-point of the fourth lidar gate (123 m a.g.l.) as a minimum value, similar to the method of Bohnenstengel et al. (2015). For two days (11 and 12 August 2012) when the lidar gate resolution was at 18 m this value was set at 63 m. Whilst this introduces a small amount of bias into the composite averages, the number of hours affected is small (13.5%). Tucker et al. (2009) found a good correlation ($r = 0.87$) during a comparison of *MH* derived using $\sigma _w^2 \left( z \right)$ with *MH* obtained from radiosonde mean temperature and relative humidity profiles.

Cloud-base height, *CB*, was estimated from attenuated aerosol backscatter profiles, $\beta$(z), measured in vertical stare mode; *CB* was defined as the height at which backscatter increased sharply such that $\beta >-2\,\hbox {dB}$, as used by Pearson et al. (2009). Since we focus on the morning development of the UBL, relatively cloud-free conditions were required, and conditions with stratocumulus cloud were avoided as this drives mixing from above (Hogan et al. 2009). The hourly *CB* values during the period 0300 to 1400 UTC were calculated, and if *CB* < 1700 m for more than 4 h during this time period, then this day was rejected as being too strongly influenced by boundary-layer clouds.

The stability of the layer between the WCC and BT Tower platforms was estimated using the bulk Richardson number, $R_{b}$, and categorized following the Blackadar planetary boundary layer parametrization scheme (Zhang and Zheng 2004), where

3 $$\begin{aligned} R_b =\frac{({g}/\theta _v) \Delta \theta _v \Delta z}{\Delta u^{2}+\Delta v^{2}}, \end{aligned}$$

where *g* is the acceleration due to gravity, ${\theta }_{v }$ is virtual potential temperature (calculated from the mean of $\theta _{v }$ at both heights), ${\Delta }{\theta }_{v}$ is the virtual potential temperature difference across a layer of thickness $\Delta$z, and $\Delta u$ and $\Delta v$ are the differences in horizontal wind components across the layer. Four stability classes are defined as (Zhang and Zheng 2004),

- $R_{b} > 0.2$—very stable layer, little turbulence exists,
- $0< R_{b} < 0.2$—damped mechanical turbulence dominates,
- $R_{b} < 0$ and ${\vert } { MH}/L {\vert } < 1.5$—forced convection, where *L* is the Obukhov length,
- $R_{b} < 0$ and ${\vert } { MH}/L {\vert } > 1.5$—free convection.

Temperature, humidity and pressure measurements from the WXT 520 weather stations were combined with wind velocities measured by the sonic anemometers.

## Results

The following sections present analysis of the UBL evolution, and test the dependence of morning-transition and rapid-growth phase characteristics on wind speed and shear in particular.

### Composite Time Series

To test whether there are significant seasonal differences in UBL evolution, composite averages are presented. The definitions of “morning-transition” and “rapid-growth” phases are discussed, and it is concluded that modified definitions are required for the UBL.

#### Heat Fluxes

Angevine et al. (2001) defined the start of the morning-transition phase as being when the surface sensible heat flux changed sign from negative to positive (“cross-over”). This definition is evaluated here for urban flux observations.

**Fig. 2:**
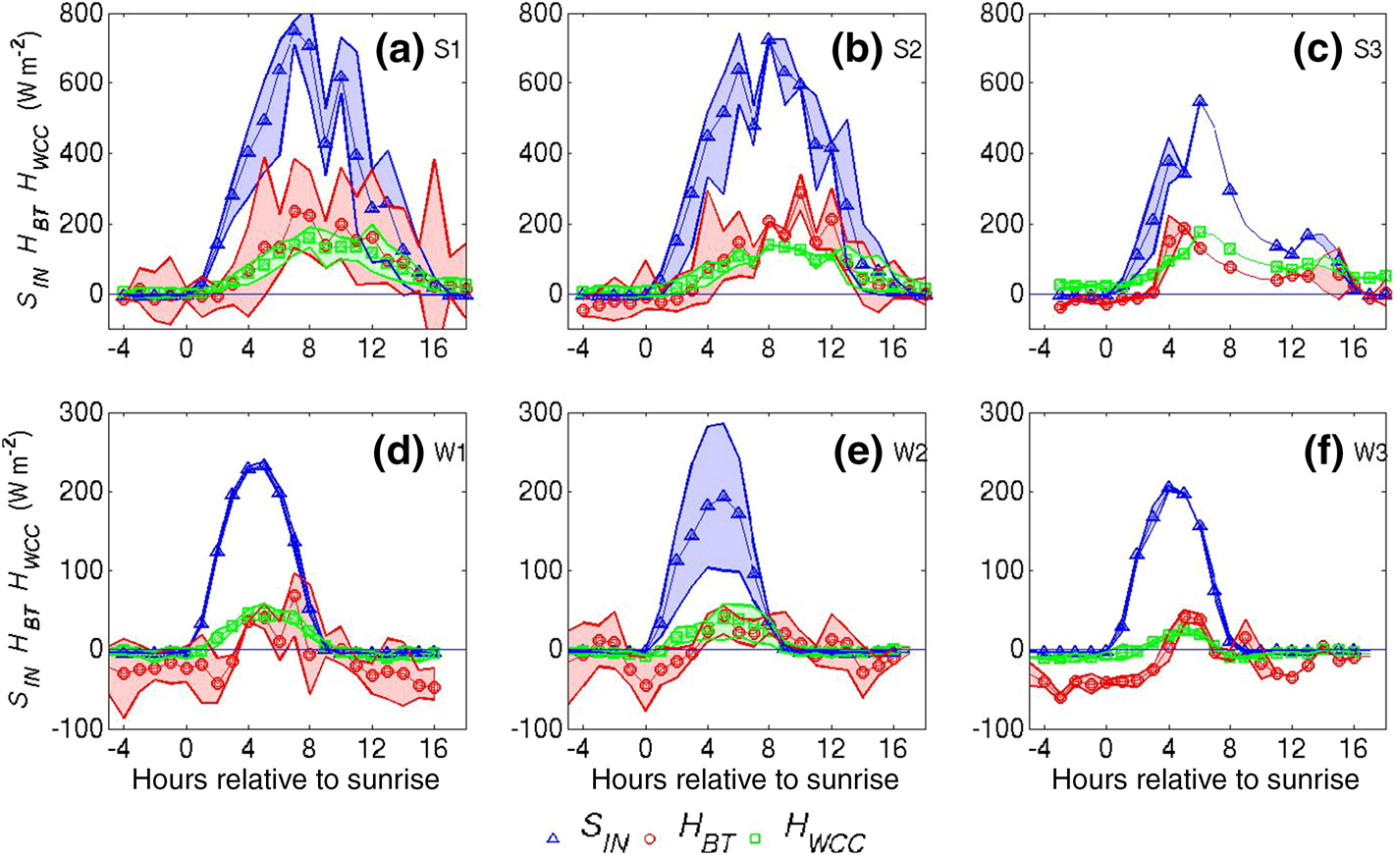
Composite plots of incoming solar radiation measured at BT*(Sin*), sensible heat flux measured at BT ($H_{BT}$) and WCC ($H_{WCC}$) for summertime (**a**–**c**) and wintertime (**d**–**f**) cases under low (**a**, **d**), moderate (**b**, **e**) and high (**c**, **f**) wind speeds. Solid lines correspond to the composite mean values and shaded areas indicate the range

Figure 2 shows that $S_{in}$ peaks later relative to sunrise in summer than in winter as days are longer at this latitude, and $S_{in}$ is almost four times higher than in winter. In summer during the day when the mixed layer is deep, $H_{WCC}$ is consistently lower than $H_{BT}$, which is explained by the WCC platform lying within the urban roughness sub-layer, as discussed in Sect. 2.2. $H_{WCC}$ is positive at night during summertime (Fig. 2a–c), as also found by Kotthaus and Grimmond (2014) for a different site in London. This result can be attributed to a combination of anthropogenic heat fluxes and large heat storage of the urban canopy. It is clear that a “cross-over” in sensible heat flux is hard to apply to urban flux measurements that have a highly “urbanized” source area.

**Fig. 3:**
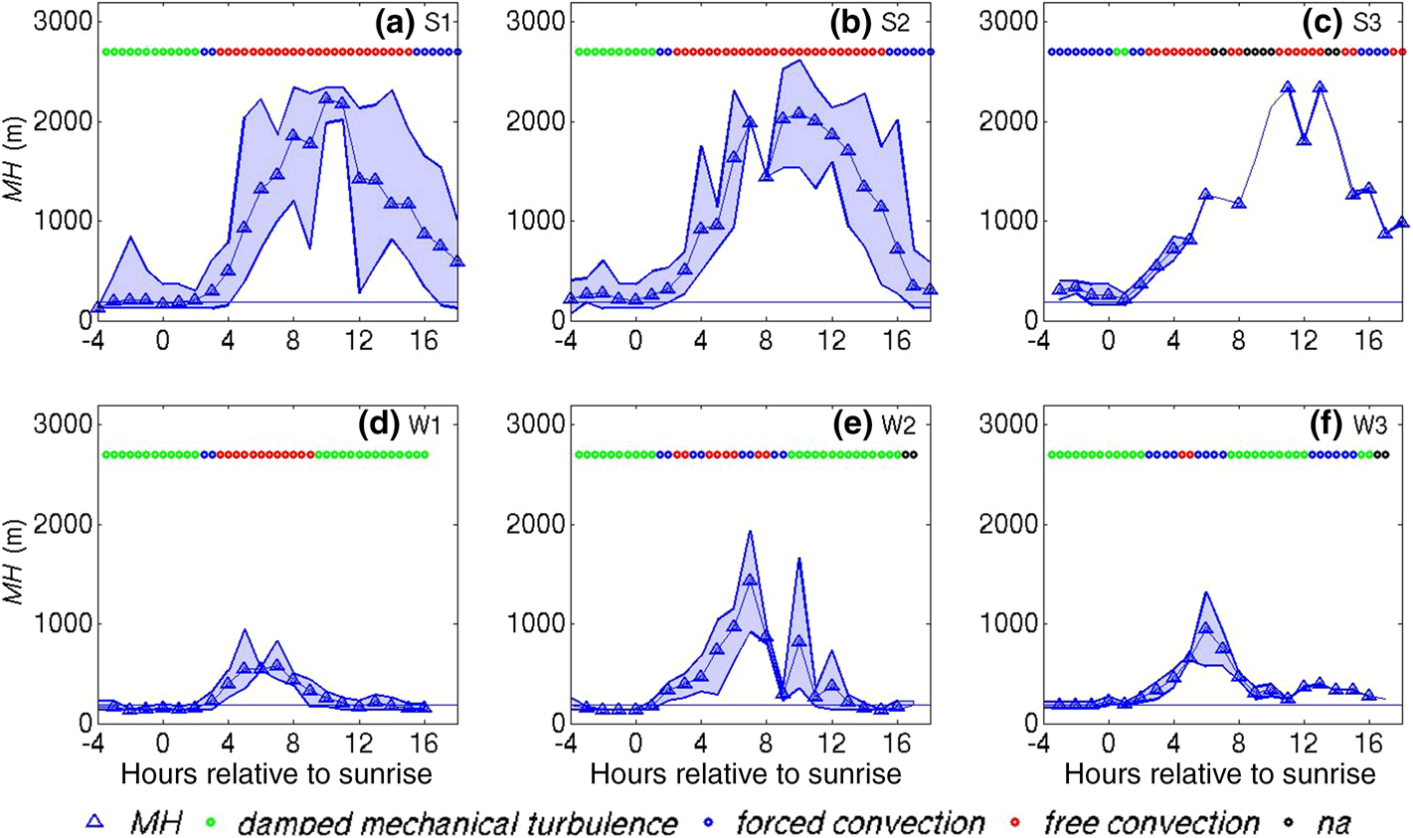
As in Fig. 2, but for mixing height (*MH*) and stability classes. Coloured markers indicate stability categories: damped mechanical turbulence ($0< R_{b} < 0.2$) forced convection ($R_{b}< 0, {\vert }{} { MH}/L {\vert } < 1.5$) and free convection ($R_{b} < 0, {\vert }{} { MH}/L{\vert } > 1.5$). na indicates cases with no data available. The reference line shows the BT Tower height

At night in the summer $H_{BT }$ is near zero or slightly negative and generally more negative than $H_{WCC}$ in both seasons. Figure  3 later shows that at night the BT Tower is near or just below the mixing-layer top in summer, and just above the mixing-layer top in winter in the residual layer, which explains the heat flux behaviour. In winter, $H_{BT}$ lags behind $H_{WCC}$ in becoming positive by 3, 2 and 1 h in the W1, W2 and W3 classes respectively, as surface-driven convection takes some time to reach 191 m in height. Hence, cross-over for $H_{BT}$ is also not meaningful in indicating the start of the morning transition. Analysis of the mixing height follows to seek an alternative definition.

#### Mixing Heights

In Fig. 3 it can be seen that *MH* is significantly lower and peaks earlier in winter than in summer, reflecting the annual cycle of thermal forcing. During the summer *MH* peaks 7 to 10 h after sunrise and the maximum mixing height *MH* $_{max} \approx 2000$ m. For winter ${ MH}_{max} < 1500$ m and is observed earlier (i.e. 5–7 h after sunrise).

Figure 3a, b, and c show that in summer at night, *MH* is near to or above the BT Tower height of 191 m, and increases with wind speed, with the mean *MH* being 190, 230 and 300 m for the S1, S2, and S3 classes respectively. In winter, *MH* is often lower than 191 m, (mean *MH* is 150, 190, and 200 m for W1, W2, and W3 respectively). Together with the heat flux behaviour, this suggests that convection often persists at night as the urban surface cools more slowly. As the urban mixing height at night is often greater than 200 m, this height cannot be used to define the time of convective onset (hereafter called the “fixed height method”), as done by Angevine et al. (2001). This implies that the onset of the rapid-growth phase should be defined differently for the UBL.

During the night, the stability class is mostly damped turbulence ($0< R_{b} < 0.2$, indicating that the layer below the BT Tower is stably stratified but turbulence is present), which persists for 1–2 h after sunrise. In summer, as wind-speed increases, night-time stability shifts to forced convection and *MH* increases. Night-time stability was never very stable (i.e. $R_{b} > 0.2$), in contrast to rural boundary layers. Angevine et al. (2001) found that the time for $R_{b} < 0.25$ (damped turbulence) to be reached was a reasonable predictor of the timing of convective onset, whereas here this stability class is already established at night. Despite differences in *MH* and stability values, convective onset is the same qualitatively: there is a significant increase in *MH* above night-time values between 1 and 3 h after sunrise, accompanied by a change in bulk stability.

**Fig. 4:**
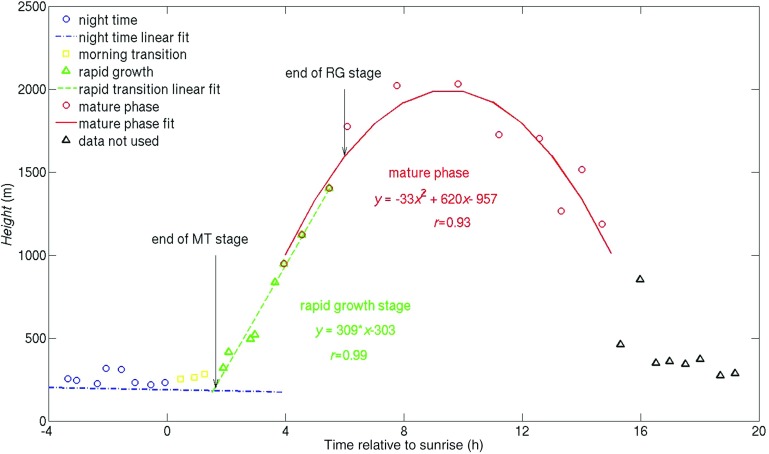
Composite plot of *MH* time series during the summer time period under moderate winds: example of the calculation of the main phases. Markers show *MH* values and lines the fits applied

### Redefining UBL Phases

Following the observations in Sect. 3.1 that, (a) urban heat flux cross-over is hard to define accurately as it is a strong function of height and source area, and (b) night-time *MH* is often greater than 200 m, the morning-transition and rapid-growth phases will now be defined by the timing of three daily events. The events are: (1) sunrise, taken here to mark the beginning of the morning transition, (2) the onset of rapid mixing-layer growth, marking the end of morning transition and the beginning of the rapid-growth phase, (3) reduced mixing-layer growth, marking the end of the rapid-growth phase and the beginning of the mature phase. Timings were calculated as follows and are illustrated in Fig. 4:

- A linear regression was applied to night-time *MH* values (i.e. all values from 0300 to sunrise, event 1).
- The end of the rapid-growth phase (event 3) was defined as the time after which *MH* varies by less than 20%. A second-order polynomial fit was applied to the mature phase mixing layer and the timing of ${ MH} = 0.8{ MH}_{max}$ defined its onset. The number of points either side of the maximum included in the polynomial fit was minimised to exclude the linear tendency in the rapid-growth period.
- Having defined the end of the rapid-growth phase, a linear fit was applied to the rapid-growth phase data points, moving backwards in time towards sunrise. The number of data points included in the fit was determined by the goodness-of-fit parameter $r^{2}$ and by examining the residuals, i.e., if points in the morning-transition phase were included in the fit, they would introduce consistently positive residuals around the regression line, and reduce $r^{2}$. The slope of the linear regression gave the rapid-growth phase growth rate, $\Delta _{MH}$.
- The intercept of the two lines (night-time and rapid growth) was considered as the end of the morning-transition phase, or “convective onset” (ii).

Figure 4 shows the method applied to the mean *MH* time series for class S2. In order to show *MH* values at higher time resolution than the hourly means presented in Fig. 3b, block averages of four points were calculated. Applying a second-order polynomial fit to *MH* around its maximum gave ${ MH}_{max} = 1900$ m, and the end of the rapid-growth phase occurred at 6 h when ${ MH} = 0.8 { MH}_{max} = 1592$ m. The intercept of the linear fit to night-time values with the linear fit to rapid-growth phase values was found at 1.65 h and marks the beginning of the rapid-growth period. Thus, in this example the morning-transition duration $T_{MT }= 1.65$ h and the rapid-growth phase duration $T_{RG }= 4.35$ h. The rapid-growth phase growth rate, ${\Delta }_{MH}= 310\,\hbox {m}\,\hbox {h}^{-1}$.

The sensitivity of the calculations to the 20% cut-off value (the percentage of *MH* variation after the rapid-growth phase) was tested by varying the value between 10 and 30%. The end of both morning-transition and rapid-growth phases and ${\Delta }_{MH}$ were calculated using a 10% cut-off value and they differed by 10, 17, and 10% from values calculated using a 20% value (mean differences of 0.5, 0.8 and 48 m h$^{-1}$). Similar differences (10, 15, 12%) were found between 20 and 30% cut-off values. Differences greater than 100% were found for two days (25 July 2011 and 13 January 2012), which were excluded from further analysis. For two more days (22 August 2011 and 22 December 2011) with uncertainties $> 50\%$ the timings were determined based on visual inspection of the *MH* and *H* time series.

Convective onset times and ${\Delta }_{MH}$ were calculated for each day. Onset times varied between 0.7 and 4 h after sunrise, the average being 2.1 h. For winter the range was smaller than summer: minimum and maximum values were respectively 0.2 and 2.9 h during winter, compared to 0.7 and 4.3 h during summer. These results are in general agreement with the values found by Angevine et al. (2001). ${\Delta }_{MH}$ varied significantly between summer and winter: minimum, maximum and mean values were 143, 885 and 400 m h$^{-1}$ during summer and 51, 420 and 160 m h$^{-1 }$ during winter, respectively.

A comparison between the convective onset time calculated with the new “slope intersection method” and the “fixed height method” used in Angevine et al. (2001) was performed for all days when night-time *MH* values were lower than 200 m. This gave a robust correlation (Spearman correlation coefficient $r = 0.73$, $p = 0.0002$), which was equally strong during summer and winter periods ($r = 0.72$, $p = 0.004$, and $r = 0.79$, $p = 0.04$, respectively), showing that on average, earlier onset times are calculated with the new method (summer slope = 0.77, offset = 0.49 h; winter slope = 0.73, offset = −0.17 h).

### Characteristic Parameters for the Morning-Transition and Rapid-Growth Phases

In Sect. 3.2 the morning-transition duration, $T_{MT}$, and rapid-growth phase growth rate $\Delta$ $_{MH}$ were introduced. For rural surfaces it was found that $T_{MT }$ and ${\Delta }_{MH}$ depend strongly on basic meteorological parameters such as wind speed and stability (e.g., Angevine et al. 2001). In the following, the dependence of these parameters on basic meteorological variables will be examined for the urban dataset.

Spearman correlation coefficients between $$T_{MT }$$ and $${\Delta }_{MH}$$ and basic meteorological parameters are presented in Table 2. The dependence of $$T_{MT}$$ on bulk Richardson number averaged over 2 h before sunrise for each day $$({ Ri}_{NT})$$ shows a moderate positive correlation ($$r = 0.38$$ for all data), indicating that the duration of the morning transition is weakly dependent on stability beforehand. In summer it is notable that slightly unstable night-time conditions (negative $${ Ri}_{NT})$$ still lead to $$T_{MT} \approx 1{-}2$$ h. The correlation is stronger in summer; in winter it is not statistically significant ($$r = 0.49$$, $$p = 0.3$$ for summer and $$r = 0.29$$, $$p = 0.56$$ in winter respectively). Angevine et al. (2001) found a relatively weak relationship with stability (determined by several parameters), and noted differences between sites—a stronger relationship was seen for a site with a wider range of values. Neither of the rural sites studied had as small a range of stability as presented here, and therefore it is noteworthy that there is any relationship with stability at this urban site.

**Table 2:** Spearman correlation coefficients (*r*) and *p* values between morning-transition duration ($$T_{MT})$$, rapid-growth rate $$({\Delta }_{MH})$$ and basic meteorological parameters: bulk Richardson number averaged over 2 h before sunrise ($${ Ri}_{NT}$$), mean wind speeds during morning-transition and night-time ($$U_{MT}$$ and $$U_{NT }$$ respectively) and convective velocity scale $$(w_*)$$ during the rapid growth period

	$T_{MT}$			${\Delta }_{MH}$		
	All data	Summertime	Wintertime	All data	Summertime	Wintertime
$R_{INT}$	0.38 (0.06)	0.49 (0.03)	0.29 (0.56)	–	–	–
$U_{MT}$	$-$0.77 (0.00)	$-$0.73 (0.00)	$-$0.68 (0.11)	–	–	–
$U_{RG}$	–	–	–	$-$0.39 (0.04)	$-$0.73 (0.00)	$-$0.68 (0.11)
$w_{*}$	–	–	–	0.79 (0.05)	0.75 (0.00)	0.57 (0.01)
${\Delta }_{MH}$	0.72 (0.00)	0.79 (0.00)	0.43 (0.35)	–	–	–

The last row presents *r* and *p* values between $T_{MT}$ and ${\Delta }_{MH}$

A stronger relationship ($r = -0.77$) is found between $T_{MT}$ and the wind speed averaged over the morning transition, $U_{MT,}$ for all data. Similar correlations are evident for both summer and winter periods ($-0.73$ and $-0.68$ respectively; statistically significant only in summer), meaning that the morning transition is shorter for higher wind speeds. As discussed in Sect. 1, similar results were also found in Angevine et al. (2001) and Beare (2008) for the time between heat flux crossover and convective onset. Given the small range of stability exhibited in these urban data, wind speed is a better explanatory variable for the length of the morning-transition period.

Table 2 also shows that mean wind speeds in successive time periods are highly correlated, i.e. a positive correlation ($$r = 0.89$$) between morning-transition and night-time mean wind speeds ($$U_{MT}$$ and $$U_{NT})$$ was found ($$r = 0.85$$ and 0.83 for summer and winter respectively) in agreement with the findings of others (e.g., Angevine et al. 2001). Also, $$U_{MT}$$ is negatively correlated with $${ Ri}_{NT}$$ ($$r = -0.62$$ and $$-0.84$$ for summer and winter respectively). Thus it can be deduced that these three parameters ($${ Ri}_{NT}, U_{MT}, U_{RG})$$, are associated with a more rapid transition to convective onset.

A statistically significant, positive dependence of ${\Delta }_{MH}$ on the convective velocity scale $w_{*}$ was found ($r = 0.79$; see Sect. 3.5.2, Eq. 6 for $w_{*}$ calculation), as might be expected. A weaker negative relationship between ${\Delta }_{MH}$ and $U_{RG}$ can be seen for all data ($r = -0.39$) that is stronger in summer ($r = -0.73$). One mechanism whereby wind can inhibit convective growth is “shear sheltering” (Hunt and Durbin 1999; Conzemius and Fedorovich 2006), where convective thermals are prevented from penetrating the capping inversion due to the deforming effect of shear across it.

**Fig. 5:**
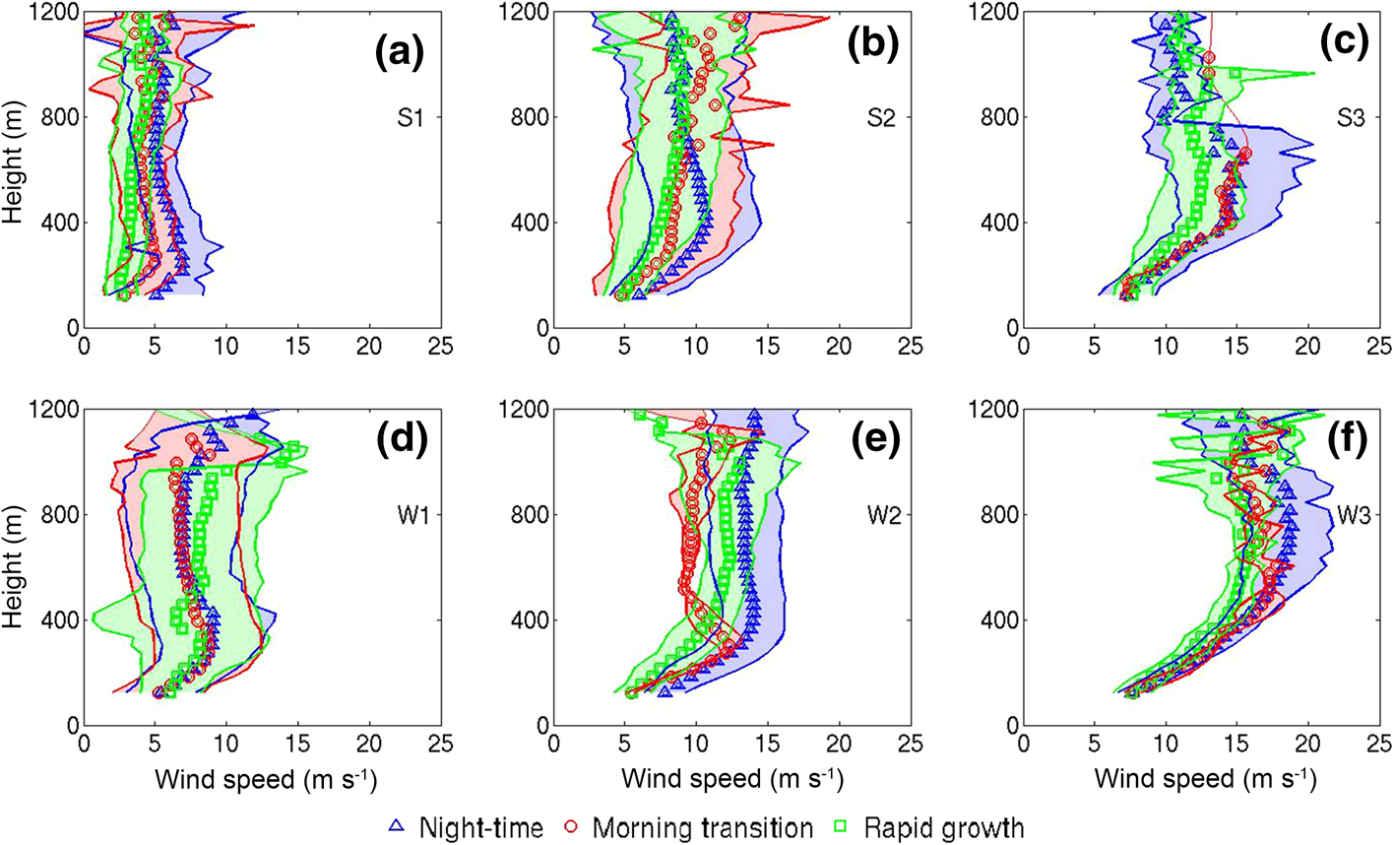
Composite vertical profiles of hourly-averaged wind speeds for summertime (**a**–**c**) and wintertime (**d**–**f**) cases under low (**a**, **d**), moderate (**b**, **e**) and high (**c**, **f**) wind speeds obtained from lidar. Points (bullets, squares, triangles) show mean values; shaded areas the range (minimum–maximum)

A positive correlation was found between $${\Delta }_{MH}$$ and $$T_{MT}$$ ($$r = 0.72$$); the correlation is stronger in summer ($$r = 0.79, p = 0.00$$) than in winter ($$r = 0.43, p = 0.35$$). This can be understood from the previous results: higher wind speeds have seemingly opposite effects in either period, being associated with earlier convective onset (smaller $$T_{MT})$$ but weaker convective growth (smaller $${\Delta }_{MH})$$. Given the demonstrated dependence of the characteristic parameters on wind speed, the next Sections explore the role of wind in UBL development.

### Composite Vertical Profiles

In this Section the variation of vertical wind-speed and turbulence profiles across the night-time, morning-transition and rapid-growth phases are examined. Discussion of boundary-layer structure in terms of vertical profiles is important to demonstrate the relationship between regional atmospheric flows and the UBL.

#### Wind-Speed Profiles and Shear

Figure 5 shows composite-averaged vertical profiles of the hourly-averaged wind speed from lidar measurements. Close to the surface, wind speeds are reduced due to surface drag, and the amount of wind shear within the UBL varies across all classes. Aloft, the profiles show that background regional flows are characterized by higher wind speeds and weaker shear. In summer and under low and moderate winds (Fig. 5a, b), a local wind-speed maximum with the form of a low-level jet (Banta et al. 2002) is observed in night-time profiles at heights of 200 m (low) or 400 m (moderate) a.g.l. with a mean wind speed at the core of the jet $$\bar{U}_{LLJ}\approx 7\,\hbox {m}\,\hbox {s}^{-1}$$ (low) and 10 m s$$^{-1}$$ (moderate). The LLJ is weaker but still observable during the morning-transition phase ($$\bar{U}_{LLJ}\approx 5$$ or 8 m s$$^{-1})$$ while in the rapid-growth phase, wind shear is weaker due to convection. In wintertime, and under low winds, LLJs are also observed. The LLJ core is situated $$\approx $$ 400 m a.g.l. and $$\bar{U}_{LLJ} \approx 9.1$$ and $$8.7\,\hbox {m s}^{-1 }$$ for the night-time and morning-transition phases respectively. Under moderate winds the LLJ is observed only during the morning transition, while under higher wind speeds, the wind speeds collapse into a more neutral profile during the morning-transition and rapid-growth phases. Less emphasis is put here on S3 profiles (Fig. 5c) due to the small number of observations (two days).

For every hour of each day, the wind shear beneath the mixing-layer top was calculated using a second order central scheme. Wind shear at the *i*th lidar gate is calculated as,

4 $$\begin{aligned} \frac{\hbox {d}\bar{U}}{\hbox {d}z_i }=\frac{\bar{U}_{i+1} -\bar{U}_{i-1} }{2\Delta _z }, \end{aligned}$$

where $\bar{U}$ is the hourly averaged wind speed and ${\Delta z}$ the gate length. Wind-shear data for all days in a class were transformed onto the timeline with respect to sunrise, and composite averages for each hour were calculated, weighted by the number of observations below $z=\textit{MH}$ for each day. From time series of the composite-averaged wind shear below $z=\textit{MH}$ (not shown) it was evident that high values of wind shear prevail throughout the night and persist throughout the morning transition, albeit reducing with time. During the rapid-growth phase when the UBL is well-mixed wind shear is smaller.

**Table 3:** Mean values and range of the absolute wind shear below $z=$ *MH* for each wind speed class and season

Class	Mean absolute shear below $z=$ *MH* (minimum–maximum) $\times \, 10^{-3}\,\hbox {s}^{-1}$		
	Night-time	Morning transition	Rapid growth
Summer			
S1	31 (12–149)	22 (2–60)	9 (3–19)
S2	28 (9–149)	24 (3–60)	15 (4–19)
S3	22 (13–29)	23 (23–23)	17 (8–29)
Winter			
W1	22 (13–36)	28 (28–28)	19 (14–40)
W2	32 (13–61)	32 (28–47)	17 (6–38)
W3	33 (23–47)	42 (38–46)	26 (14–40)

Table 3 shows the average absolute wind shear $\frac{\hbox {d}\bar{U}}{\hbox {d}z}$ below $z=\textit{MH}$ for all wind speed classes in summer and winter for each phase. Absolute wind shear values were calculated to account for changes of wind shear sign within the UBL. For this calculation, hourly-mean values of wind shear were averaged over all heights below $z=\textit{MH}$ within each phase, weighted by the number of observations for each phase. In general, wind shear is higher during the night-time and morning-transition phases than the rapid-growth phase for all classes. During the rapid-growth phases the wind shear is smallest as convective mixing prevails. This is consistent with the simulations of Beare (2008), where the importance of wind shear during the night-time and morning-transition phases was highlighted, in contrast to the well-developed mixed-layer where buoyancy is dominant. The contribution of shear in the development of turbulence will be explored in Sect. 3.6.

**Fig. 6:**
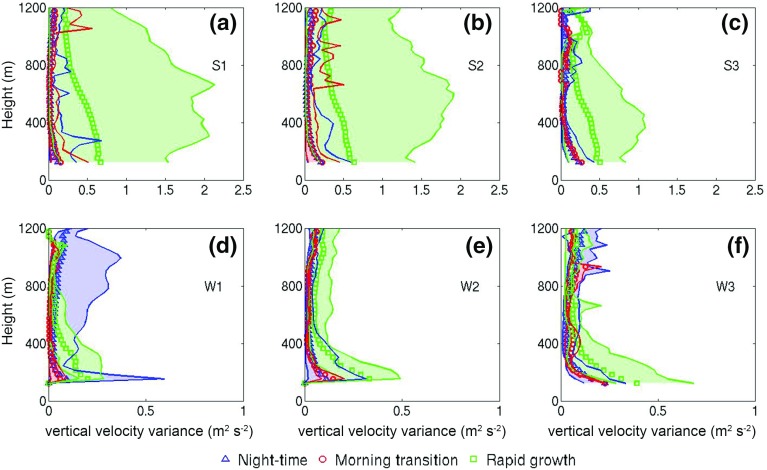
As in Fig. 5 but for profiles of the vertical velocity variance

#### Vertical Velocity Variance Profiles

Figure 6 shows that $\sigma _w^2$ is largest in the rapid-growth phase for all wind-speed classes: maximum composite averaged ${\overline{\sigma _w^2} }$ values range from $\approx 0.67\, \hbox {m}^{2}\,\hbox {s}^{-2}$ (S1) to $\approx 0.34\,\hbox {m}^{2}\,\hbox {s}^{-2}$ (W1). It can be seen that ${\overline{\sigma _w^2} }$ decreases across wind speed classes as $S_{IN}$ decreases (See Fig. 2). A marked difference is evident in $\sigma _w^2$ profiles for different phases: during night-time and morning-transition phases $\sigma _w^2$ decreases monotonically with height, whilst rapid-growth phase profiles resemble classical CBL profiles with an elevated maximum. This is evident during the summertime, under low or moderate winds, and can be seen more clearly from the maximum values. As wind speed increases, profiles resemble neutral-condition profiles (S3, W2 and W3 classes, Fig 6c, e, f). The $\sigma _w^2$ profiles and their dependence on flow structure and surface forcing will now be explored in more detail.

### Scaling Wind-speed and $\sigma _w^2$ Profiles: Regional Flows Versus Surface Forcing

Identifying appropriate scaling for meteorological profiles is important because it helps to improve model parametrizations and basic understanding of atmospheric processes (Barlow 2014). Banta et al. (2006) explored scaling of night-time wind speed and turbulence profiles to test whether turbulence was generated at the surface and transported upwards (“traditional” boundary layer), or generated by a primary source aloft and transported downwards (“upside down” boundary layer). For daytime unstable conditions, Wood et al. (2010) and Barlow et al. (2011a) found that mixed-layer scaling was appropriate for $\sigma _w^2$ data measured over London. In this section, the significance of regional flows (i.e. LLJs) versus surface processes in generating turbulence and shear is tested by scaling the wind speed and turbulence profiles in all phases. The low wind-speed, summertime class (S1) was selected since substantial radiative forcing, large surface heat fluxes, large mixing height (Figs. 2a, 3a) and LLJs (Fig. 5a) are all present. Also, this class has the most hourly observations (191 h) across the largest number of days (11 days).

#### Night-Time and Morning-Transition Profiles: LLJ Scaling

LLJs over the urban surface have been often reported (e.g. Wang et al. 2007; Kallistratova and Kouznetsov 2012; Barlow et al. 2015), but their interaction with the boundary-layer structure is not yet fully understood (Klein et al. 2016). Banta et al. (2006) studied night-time LLJs and found that wind-speed and turbulence profiles scale better with the wind speed at the core of the jet ($$U_{LLJ} )$$. A similar approach was proposed in Smedman et al. (1993), with the addition that heights were scaled with a length scale characteristic of the jet’s dimension.

**Fig. 7:**
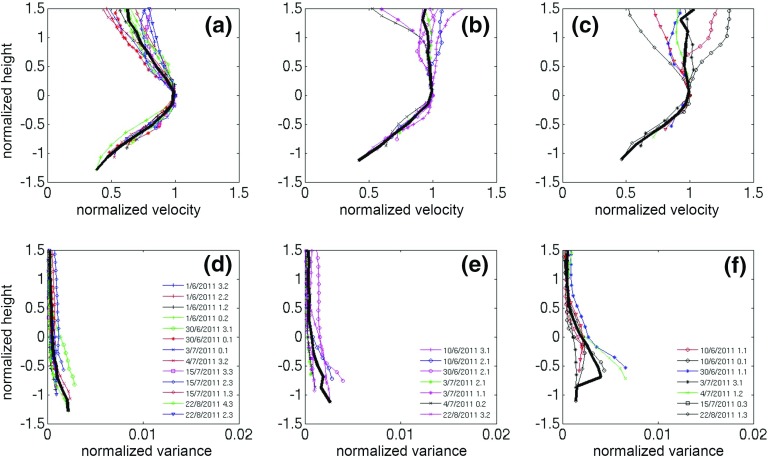
Hourly-averaged vertical profiles of normalized wind speeds $$U/{U_{LLJ} }$$—**a**–**c** ($$U_{LLJ} $$ is the wind speed at the core of the jet) and vertical velocity variance $${\sigma _w^2 }/{U_{LLJ}^2 }-\hbox {d},\hbox {e},\hbox {f}$$ versus normalized heights $$h/{d_{LLJ} }$$ ($$d_{LLJ}$$ is the jet’s half width) for summertime cases and under low wind speeds during the night-time phase. **a, d**: cases with a distinct maximum in the wind-speed profile and $$\sigma ^2_w$$ profiles monotonically decreasing; **b, e**: cases with logarithmic-like wind-speed profile and $$\sigma ^2_w$$ profiles monotonically decreasing, and **c, f**: cases with $$\sigma ^2_w$$ profiles having a maximum aloft, and wind-speed profiles with either LLJ like or logarithmic-like profiles. Bold black lines are composited mean values. Legends show the time stamp of every line in the format day/*month/year hour before sunrise*

Here, following Banta et al. (2006), wind speed and $$\sigma _w^2 $$ profiles are scaled by using $$U_{LLJ} $$ (and $$U_{LLJ}^2$$), defined as the first wind-speed maximum above the surface. After several length scales were tested, it was decided to scale heights by the length scale $$d_{LLJ} ,$$ which is the jet’s half width, i.e. the distance below the jet where $$U=1/2U_{LLJ} $$, a scale used in the self-similarity approach for round jets (Pope 2000). For every wind-speed profile, a second-order polynomial curve fit was applied to the wind speeds measured in the four lidar gates closest in height to $$U_{LLJ} $$. With the normalization used here, the jet maximum is located at the points (1,0) (Fig. 7).

Three categories were created based on the shape of the wind-speed and variance profiles, similar to Banta et al. (2006):

1. the wind-speed profile has a distinct maximum (LLJ) and the $\sigma _w^2$ profile decreases monotonically,
2. the wind-speed profile is logarithmic and the $\sigma _w^2$ profile decreases monotonically,
3. $\sigma _w^2$ profiles have a maximum aloft, and the wind-speed profile is either LLJ-like or logarithmic-like.

It should be recalled that the lidar cannot observe close to the ground ($z < 124$ m), which introduces some uncertainty, as the $\sigma _w^2$ profiles are hard to categorise due to the maximum not always being fully resolved. For the wind speed profile on 30 June 2011 at 0.1 h before sunrise, multiple maxima of similar strength were located close to each other, and the height of the LLJ was defined by visual inspection to be 394 m. One $\sigma _w^2$ profile (5 July 2011 at 3.2 h before sunrise) was too noisy and was excluded from the analysis.

**Fig. 8:**
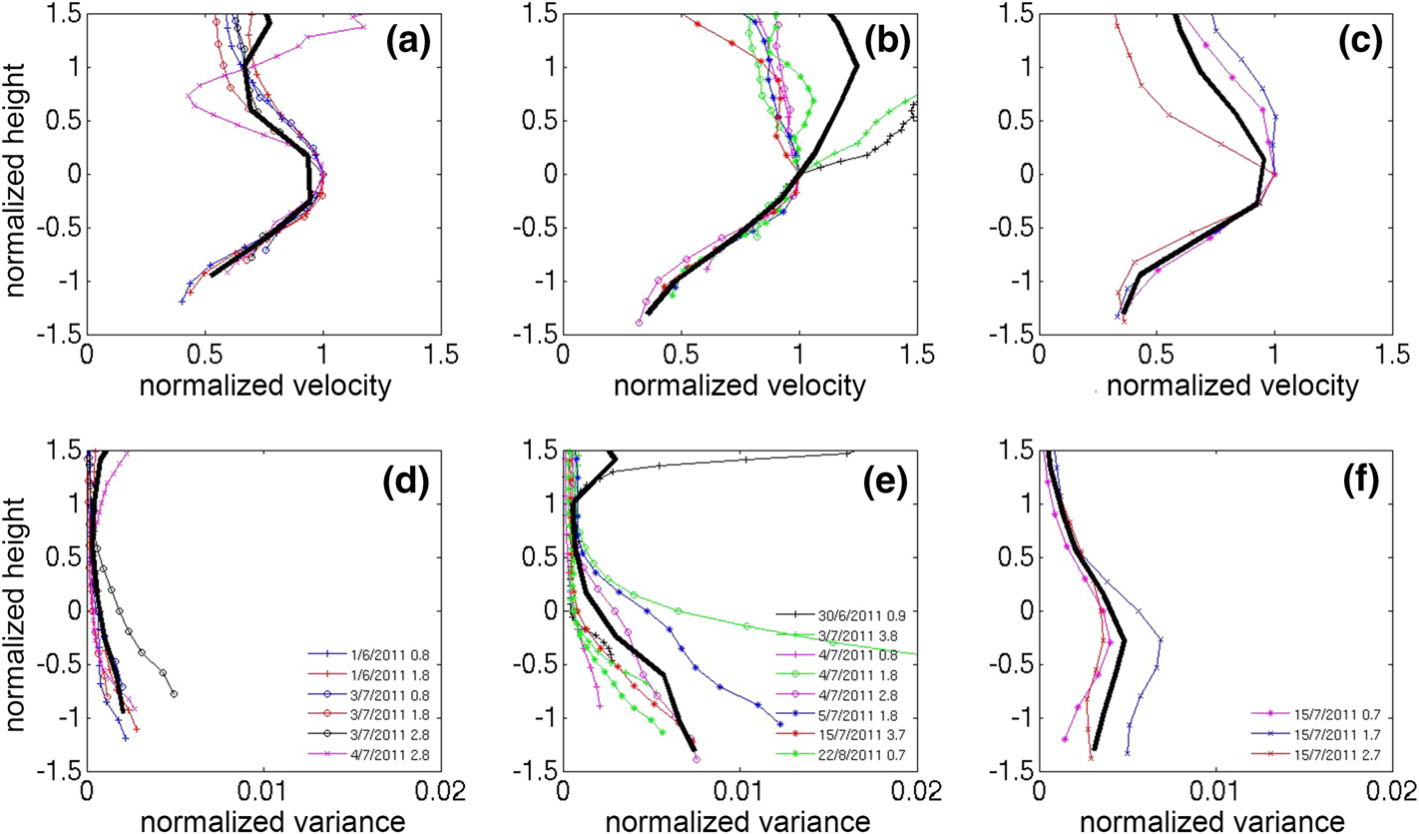
As in Fig. 7 but for the morning-transition phase

Figure 7 shows the scaled *U* and $$\sigma _w^2 $$ profiles for the night-time phase for class S1. For each category, the scaling causes *U* profiles to collapse nicely, especially below the jet height ($$z/d_{LLJ} =0)$$. $$\sigma _w^2 $$ profiles show more spread around the mean. The peak of the ratio $${\sigma _w^2 }/{U_{LLJ}^2 }$$ is of the same magnitude for the first two categories (0.003) and slightly higher for category 3 (0.004). Both results were also found in Banta et al. (2006), where successful collapse using LLJ scaling below jet height was interpreted to mean that the boundary layer was “upside-down” and turbulence was dominated by downward transport from the jet.

LLJ scaling is moderately successful for morning-transition profiles (Fig. 8), even though some discrepancies do occur. For example, the profile observed at 1.8 h after sunrise on 4 July 2011, which deviates significantly from the mean $\overline{{\sigma _w^2}}$ profile, occurs under unstable conditions ($R_{b }= -0.009$, free convection category). It was hypothesized that discrepancies from mean $\overline{{\sigma _w^2 }}$ profiles could be attributed to the active role of surface-generated turbulence. For both night-time and morning-transition profiles, mixed-layer scaling was also examined, testing several combinations of friction and convective velocity scales ($u_*$ and $w_*$ respectively, see Sect. 3.5.2 for details). The collapse of the resulting profiles was significantly poorer than that obtained with LLJ scaling, demonstrating the importance of turbulence generated aloft in the early phases of UBL evolution. These results are consistent with Barlow et al. (2015), who found “upside-down” turbulence characteristics in the morning phase of the UBL for a 5-day case study.

#### Rapid-Growth Profiles: Mixed-Layer Scaling

As can be seen in Figs. 5 and 6, the rapid-growth phase wind speed and turbulence profiles are similar to classical CBL profiles and therefore mixed-layer scaling was used. Heights were normalized using the mixing height *MH*, and the mixed velocity scale $$w_{m}$$ proposed in Moeng and Sullivan (1994) was used. In order to take into account both mechanical and thermal forcing at the surface, $$w_m $$ is a function of the surface friction ($$u_{*0})$$ and convective velocity scales,

5 $$\begin{aligned} w_m^3 =w_*^3 +5u_{{*}o}^3 . \end{aligned}$$

The convective velocity scale is defined as

6 $$\begin{aligned} {w_*} = {\left( {\frac{g}{{{T_o}}}{{\overline{w'T'} }_o}{z_i}} \right) ^{1/3}} \end{aligned}$$

where $z_{i}$ is the inversion height (taken here to equal *MH*), the absolute surface temperature $T_{o}$ has been estimated as the mean of BT and WCC temperatures, and surface heat flux (${\overline{w'T'}_o})$ is extrapolated from the BT Tower measured heat flux ${\overline{w'T'}_H})$ to the ground, using the relationship (Wood et al. 2010),

7 $$\begin{aligned} {\overline{w'T'}_H} = {\overline{w'T'}_o}\left( {1 - 1.2\frac{z}{{{z_i}}}} \right) . \end{aligned}$$

The surface friction velocity $$u_{*}o $$ was also extrapolated from BT Tower $$u_*$$ as,

8 $$\begin{aligned} u_*=u_{*}o \left( {1-\frac{z}{z_i }} \right) . \end{aligned}$$

The wind-speed profiles were normalized with the wind speed measured at the lowest observable height ($$U_{lower} $$ measured at $$z =124$$ m) and the $$\sigma _w^2 $$ profiles by $$w_m^2 $$.

Each rapid-growth hourly profile falls into one of two categories based on the shape of $\sigma _w^2$:

1. monotonically decreasing $\sigma _w^2$ profile,
2. $\sigma _w^2$ profile has a maximum aloft.

**Fig. 9:**
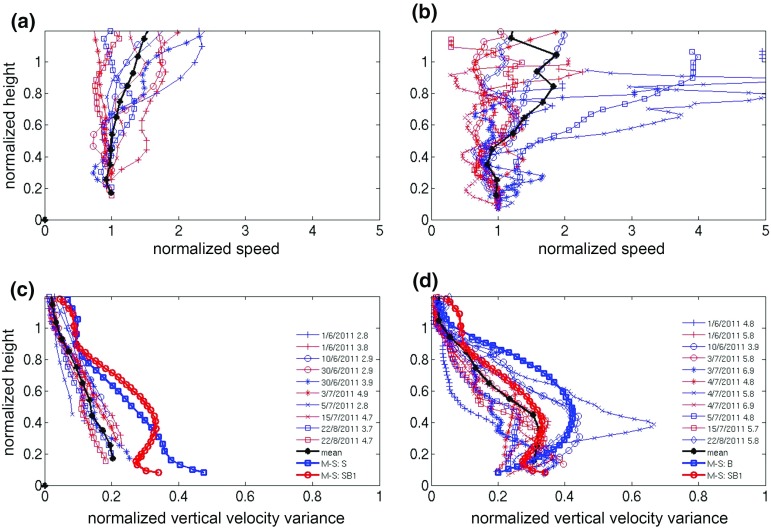
Hourly-averaged vertical profiles of normalized wind speeds $$U/{U_{lower} }$$—**a**, **b** ($$U_{lower} $$ is the wind speed at $$\approx $$ 124 m a.g.l.) and vertical velocity variance $${\sigma _w^2 }/{w_m^2 }$$—**c**, **d** ($$w_{m}$$ is the convective velocity scale) for summertime cases under low wind speeds during the rapid-growth phase. **a**, **c**: cases with monotonically decreasing $$\sigma _w^2 $$ profile; **b**, **d**: cases where $$\sigma _w^2 $$ profiles have a maximum aloft. Bold black lines are composited mean values. Also shown are three theoretical profiles obtained from LES runs (Moeng and Sullivan 1994): a simulation with shear—dominated flow with no surface flux—*S* (**c** blue squares), a simulation with a buoyancy—dominated flow with a small shear effect—*B* (**d** blue squares), and an intermediate flow with strong shear and moderate convection—SB1 (**c**, **d** red circles). Legends show the time stamp of every line in the format day/*month/year hour after sunrise*

Results of the mixed-layer scaling for $\sigma _w^2$ are shown in Fig. 9c, d. Three profiles obtained from LES runs (Moeng and Sullivan 1994) are superimposed for buoyancy-dominated (*B*) and shear-dominated (*S*) flows, and an intermediate flow with strong shear and moderate convection (*SB*1). Profiles in category 1 collapse reasonably well onto a monotonically decreasing profile (Fig. 9c). The shape is similar to the S profile, but at considerably lower values. On the other hand, profiles of category 2 collapse to a mean profile that closely resembles the SB1 profile. The mean $\overline{{u_*}/{w_*}}$ ratio is significantly higher for category 1 ($\overline{{u_*}/{w_*}} \approx 0.58$ and 0.35 for categories 1 and 2 respectively), confirming the predominance of shear processes.

As can be seen from the legends in Fig. 9, profiles in category 2 occur later in the rapid-growth phase, whilst category 1 profiles occur earlier. It should be noted that using LLJ scaling for the rapid-growth profiles (not shown) gives poor results. Even though there is significant spread across the hourly wind-speed profiles, it is apparent that under both categories, mean wind speed ($\overline{U/{U_{lower} }})$ profiles are similar (Fig. 9a, b) with increased shear in the upper part of the UBL.

### Analysis of Turbulent Kinetic Energy Production in the UBL

It has been established thus far that aside from surface sensible heat flux, wind speed also plays a role in determining (a) the duration of the morning transition, and (b) the rapid-growth rate of the UBL. Additionally, there is a significant level of wind shear present during most phases of the UBL, therefore its evolution is not governed by buoyant processes alone. In this section, an attempt is made to quantify both shear and buoyant production of turbulent kinetic energy (TKE) within the UBL, using previously established theoretical frameworks.

#### Shear Production of TKE Within the Rapid-Growth Phase

It is well known that in the CBL, growth is driven by the surface buoyancy flux, and that entrainment by overshooting thermals enhances the growth rate (e.g. Carson 1973). In some theoretical studies of the CBL with shear (e.g. Pino et al. 2003; Conzemius and Fedorovich 2006; Fedorovich and Conzemius 2008), it has been argued that TKE production by shear across the entrainment zone contributes to entrainment; and that shear production at the surface is dissipated locally and is not transported upwards to contribute to entrainment. Given that the measurements were made over an urban surface with large roughness length, it is hypothesized that surface shear production may be comparable to elevated shear production.

To address this issue, calculations were made based on the zero-order model representation of surface ($$S_{0})$$ and boundary-layer top ($$S_{Zi})$$ shear contributions to the integral TKE budget, as defined by Fedorovich (1995) (see Appendix),

9 $$\begin{aligned} S_0= & {} u_m \tau _{xs} +v_m \tau _{ys} , \end{aligned}$$

10 $$\begin{aligned} S_{z_i }= & {} \frac{1}{2}\left( {\Delta u^{2}+\Delta v^{2}} \right) \frac{\hbox {d}z_i}{\hbox {d}t}, \end{aligned}$$

where $$u_m $$ and $$v_m $$ are the vertically-averaged horizontal velocity components, $$\tau _{xs} $$ and $$\tau _{ys} $$ are the components of the surface stress, $$\Delta u$$ and $$\Delta v$$ are the jumps across the inversion of the horizontal velocity components, and $$\frac{\hbox {d}z_i}{\hbox {d}t}$$ is the rate of change of the inversion height. In the present study, where a streamwise rotation has been applied to wind-speed data, the surface and boundary-layer top shear generation terms have been approximated to become $$S_0 =u\tau _{xs} $$ and $$S_{MH} =\frac{1}{2}\Delta u^{2}\frac{\hbox {d}MH}{\hbox {d}t}$$ where $$\Delta u$$ is the jump of the streamwise wind speed measured with the lidar for gates immediately above and below $$z =$$ *MH*, $$\tau _{xs} $$ is the surface streamwise momentum flux and *u* the streamwise component of the wind speed. The values used were the wind speed and the streamwise stress at the BT Tower, the latter being extrapolated to the ground according to the method given in Brost et al. (1982). $$\frac{\hbox {d}MH}{\hbox {d}t}$$ was estimated by applying a fourth-order polynomial fit to the *MH* time series. As discussed in Conzemius and Fedorovich (2006), different authors interpret jumps differently and hence the values here may not be directly comparable with other studies—here the emphasis is on the relative size of the terms.

In Table 4 the median values of $$S_{0}$$ and $$S_{MH}$$ are presented for the rapid-growth phase in all wind speed classes. Because the method is valid only under the assumption of a uniform wind speed profile within the boundary layer, night-time and morning-transition phases were excluded from this analysis, as it was shown in Table 3 that large shear exists below $$z=$$ *MH* for these phases. For the same reason, only hours with relatively low wind shear (i.e. $$S < 0.016\,\hbox {s}^{-1})$$ were used. Table 4 shows that median $$S_{MH} > S_{0}$$ for all wind speed classes except W2. The relative magnitude of boundary-layer top to surface shear production (calculated as $$S_{MH}/S_{0})$$ varies between 11 (S1) and 0.8 (W2), the average in each season being 5.8 in summer and 1.5 in winter. These results show that shear production near the top of the UBL is larger than surface shear production, despite large surface roughness, and that this is more pronounced in summer rather than winter.

**Table 4:** Median values of the surface ($$S_{0})$$ and ABL top ($$S_{MH})$$ shear contributions to the integral TKE budget for the rapid-growth phase with low shear, calculated with a zero-order model representation

	$S_{MH}~(\hbox {m}^{3}\,\hbox {s}^{-3})$	$S_{0}~(\hbox {m}^{3}\,\hbox {s}^{-3})$
Summer S1	2.2 (17.0)	0.2 (1.7)
Summer S2	3.5 (6.7)	1.3 (1.0)
Summer S3	9.8 (45.6)	2.6 (2.8)
Winter W1	5.1 (2.7)	4.4 (12.9)
Winter W2	2.1 (42.6)	2.7 (1.2)
Winter W3	12.3 (0.8)	5.1 (0.2)

Values in parenthesis are standard deviations

#### Shear and Buoyancy Terms of the Local TKE Budget

In Sect. 3.4 it was shown that shear can be relatively large in the night-time and morning-transition phases. To examine the contribution of shear to mixing, relative to buoyancy, in this section the shear and buoyancy production terms of the local TKE budget will be estimated for the BT Tower during different phases of UBL development. Only the low wind-speed class in summer (S1) will be analysed, for the reasons given in Sect. 3.5.

The buoyancy and shear production terms of the TKE budget are defined as $g /{\theta _v}\overline{w'\theta _v^{'}}$ and $\overline{u'w'}{\vartheta \bar{U}}/{\vartheta {z}}$ respectively, where $\overline{{w}'{\theta } _{v}{'}}$ is the turbulent heat flux, $\overline{u'w'}$ is the streamwise momentum flux and ${\vartheta \bar{U}}/{\vartheta z}$ is the shear in a steamwise coordinate system (Stull (1988)). The buoyancy term and momentum fluxes at the height of the BT Tower were directly estimated from the sonic anemometer measurements; the shear term ${\vartheta \bar{U}}/{\vartheta z}$ was estimated from the difference in velocity magnitude between lidar gates that were just below and above the BT Tower, i.e. ${\vartheta \bar{U}}/{\vartheta z}\approx {\Delta \bar{U}}/{\Delta z}$. A steamwise rotation was applied to the lidar *u*, *v* and *w* wind-speed components obtained from the DBS method, using the double-rotation method (Wilczak et al. 2001), so that the mean rotated vertical and spanwise wind-speed equal zero for each hourly averaging period.

**Fig. 10:**
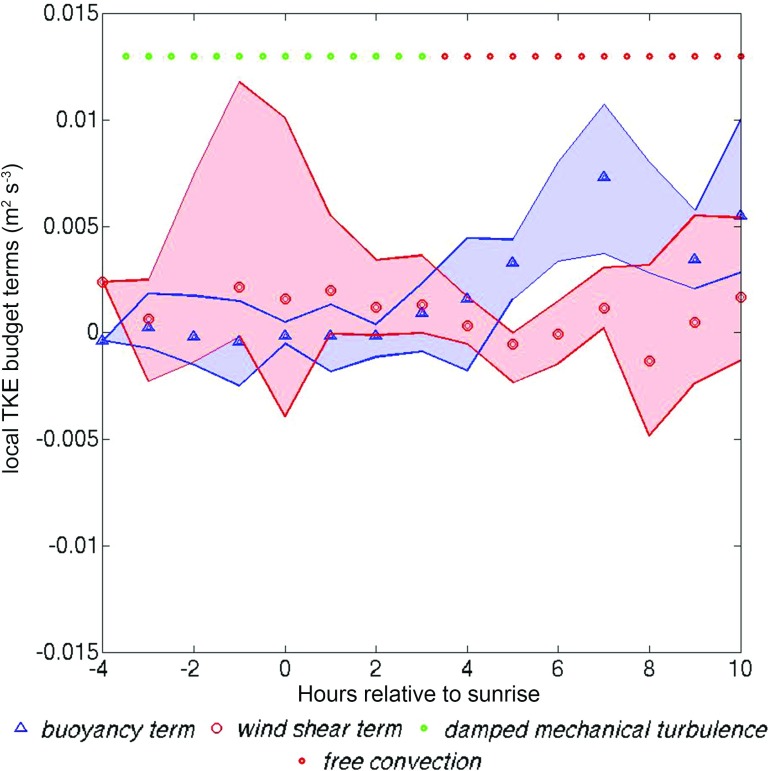
Composite plot of wind shear and buoyant production local TKE budget terms estimated at the height of the BT Tower. Points show composited mean values and shaded areas the range. Coloured markers indicate stability categories: green for damped ($0< R_{b} < 0.2$) and red for free convection ($R_{b} < 0, { {\vert }MH}/{ L{\vert } }> 1.5$)

In Fig. 10 the interplay between the thermal and mechanical production of turbulence can be seen: during the night-time and morning-transition periods, high shear production values are observed (average $\approx 0.0027$ and $0.0025\,\hbox {m}^{2}\,\hbox {s}^{-3}$ respectively). The buoyancy term is slightly negative or near-zero during these periods ($\approx -5\times 10^{-5}\,\hbox {m}^{2}\,\hbox {s}^{-3})$, when the BT Tower is near the top of the mixed layer. During the rapid-growth period (onset is identified at $\approx$ 2 h after sunrise for this wind-speed class) shear production gradually reduces, and eventually levels off to values close to zero (average $\approx 0.0003\,\hbox {m}^{3}\,\hbox {s}^{-3})$. At the onset point, the buoyancy production term increases and by 4 h after sunrise it dominates over shear production. Shear production of TKE is always positive. Negative values are not physically interpretable.

**Fig. 11:**
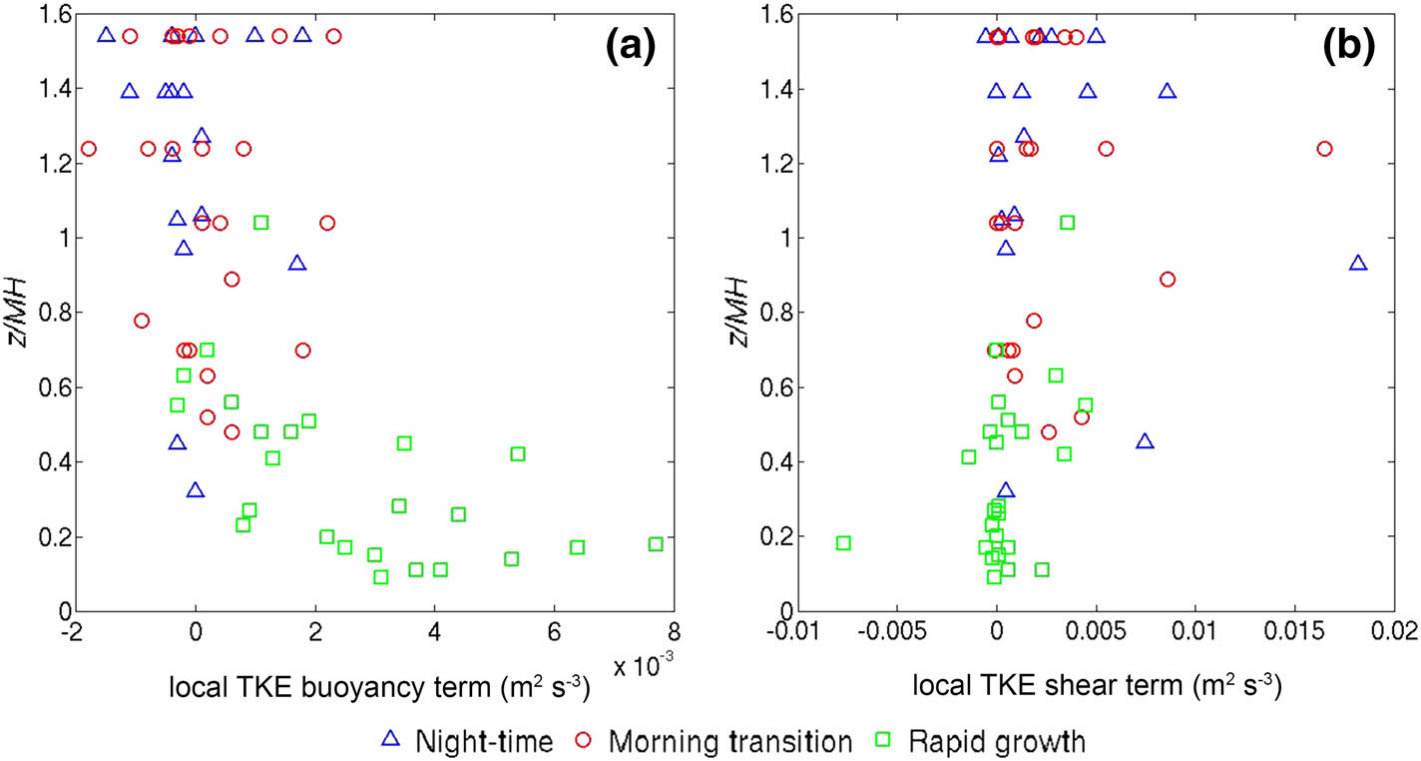
Vertical profiles of the hourly values of the **a** buoyancy and **b** shear production terms of the local *TKE* budget at the height of the BT Tower during night-time, morning-transition and rapid-growth phases for summertime cases under low wind speeds (S1). The vertical axis is the BT Tower observation height normalized by MH

As the height of the BT Tower observation relative to the mixing-layer top is changing throughout these periods, Fig. 11 shows the hourly values of the local shear and buoyancy TKE budget terms using $z_{BT }/{ MH}$ for the vertical axis normalization.

For the rapid-growth phase, the vertical profile of the buoyancy term (Fig. 11a) broadly agrees with the classical mixed-layer buoyancy profile: high values are observed near the surface, decreasing with height. Near-zero values of the buoyancy term can be observed during the night-time and morning-transition phases, when the BT Tower is just below or above $z=$ *MH*, which leads to wide scatter between negative and positive values.

Figure 11b shows that small shear term values are observed within the UBL during the rapid-growth phase: some larger values occur towards the bottom and the top, which is consistent with the TKE profiles of Conzemius and Fedorovich (2006) for a sheared CBL. The height of minimum shear production ($z_{BT}/{ MH} \approx 0.3$) is similar. During the night-time and morning-transition phases shear production is mostly positive and sometimes significantly enhanced near $z=$ *MH*, and thus much larger than the buoyancy term. Clearly there is a lot of scatter in the data, but they suggest that even under low wind speeds, shear production of TKE cannot be neglected.

## Conclusions

This study presents an analysis of the evolution of the UBL using a novel combination of ground-based remote sensing and in situ instrumentation. For a site in central London, UK, the full evolution of the UBL was captured from night-time, through the morning-transition and rapid-growth phases, until the fully-developed phase. From a long database, only cases with small amounts of boundary-layer cloud were selected. Days were classified according to wind speed (low, moderate or high), and taken from either summer or winter months, given the latitude of the site.

The night-time UBL was occasionally weakly convective, in contrast to the rural ABL, which is typically stable. Its mean depth across the wind speed classes varied between 190–300 m in summer, and 150–200 m in winter. This contrasts with the study of Angevine et al. (2001) who found night-time rural mixing heights to be less than 200 m in summer. In the present study 13.5% of the hourly *MH* values were lower than 124 m.

Due to the differing night-time conditions, in order to study the evolution of the UBL two phase redefinitions were required: (1) the start of the morning-transition phase was taken as sunrise rather than sensible heat flux cross-over from negative to positive. This was done as the urban night-time sensible heat flux was sometimes already positive, dependent on the height of the observation. (2) The end of the morning-transition phase was defined by a sharp, quasi-linear increase in *MH*. This definition resulted in morning-transition durations that were on average slightly shorter than when calculated using the Angevine et al. (2001) constant threshold of 200 m, but of a similar magnitude.

The urban morning-transition phase varied in length between 0.5 and 4 h. It had a weak, positive relationship with night-time stability (defined by the bulk Richardson number across the lowest 191 m), and a stronger negative relationship with wind speed. This held for both seasons, and is consistent with previous work (Angevine et al. 2001; Beare 2008).

The growth rate of the mixing layer during the rapid-growth phase had a strong positive relationship with the convective velocity scale, and a weaker, negative relationship with wind speed. The growth rate also had a positive correlation with the morning-transition phase duration, particularly in summer. This result highlighted the opposite effects of wind-speed, namely that increased wind speed shortens the morning-transition phase, yet reduces growth rate in the rapid-growth phase.

Given the complex dependence of UBL evolution on wind shear, vertical profiles of wind speed were more closely examined. Night-time and morning-transition wind-speed profiles were dominated by LLJs, resulting in monotonically decreasing $\sigma _w^2$ profiles, whilst during the rapid-growth phase intense turbulent mixing resulted in well-mixed wind-speed profiles. Absolute values of wind shear were highest in the night-time and morning-transition phases and lowest in the rapid-growth phase—these differences reduced with increased wind speed, and were less distinct in winter. These results are consistent with previous simulations (Beare 2008), highlighting that the morning-transition phase can be highly sheared, and its evolution sensitive to the magnitude of wind shear.

Wind speed and turbulence profiles during the night-time and morning-transition phases for the low wind speed class in summer were analysed using LLJ scaling. The resulting good collapse of profiles below the local wind-speed maximum indicated that in these phases the UBL is mostly dominated by downward transport of LLJ-generated turbulence aloft. Mixed-layer scaling during the rapid-growth phase was successful using a combination of friction and convective velocities, suggesting dominance by surface forcing.

Shear production was greater than buoyant production at night-time and in the morning-transition phase near the top of the UBL for the low wind speed class in summer. In the rapid-growth phase, buoyant production dominated at the surface, but shear production dominated in the upper half of the UBL. This is consistent with previous simulations of the sheared CBL (Conzemius and Fedorovich 2006). The results suggest that shear processes cannot be neglected in the UBL, even in cases with low wind speed.

In terms of the production of TKE by shear, even in the rapid-growth phase when mean wind shear values within the UBL were small, there was significant shear production near the boundary-layer top and at the surface. Shear production near $z=$ *MH* was around six times larger than surface shear production in summer, and around 1.5 times larger in winter. Despite the large, urban surface roughness, these results suggest that elevated shear is important in governing mixing within the UBL, and that the effect can be more pronounced in summer. In general, these results imply that regional flows such as LLJs play an important role alongside surface forcing in determining UBL structure and growth.

## Appendix

The CBL grows due to a mixture of processes, including surface-driven buoyancy, shear, horizontal convergence and heat advection. Entrainment of heat from above the growing boundary layer is a key process that has been often parametrized (see Conzemius and Fedorovich 2006 for a nice review and test of parametrizations). Given that vertical gradients of wind speed and potential temperature are negligible throughout most of the depth of the CBL, simple bulk models have been developed to describe the growth of the horizontally-homogeneous CBL by vertically integrating the Reynolds-averaged Navier Stokes equations over the depth of the CBL.

In this context, Fedorovich (1995) derived a so-called slab, or zero-order model, of the CBL. Entrainment processes, and thus the growth of the CBL, were evaluated by deriving the integral TKE budget equation,

11 $$\begin{aligned} \frac{\hbox {d}}{\hbox {d}t}\mathop \int \limits _0^{z_i } e\hbox {d}z=u_m \tau _{xs} +v_m \tau _{ys} +\frac{1}{2}\left( {\Delta u^{2}+\Delta v^{2}} \right) \frac{\hbox {d}z_i }{\hbox {d}t}+\frac{z_i }{2}\left( {B_s -\Delta b\frac{\hbox {d}z_i }{\hbox {d}t}} \right) -{\varPhi }_i -\mathop \int \limits _0^{z_i } \varepsilon \hbox {d}z\qquad \end{aligned}$$

where $u_{m}$ and $v_{m}$ are the vertically-averaged horizontal velocity components, ${\tau }_{xs}$ and ${\tau }_{ys}$ are the components of the surface stress, $\Delta u$ and $\Delta v$ are the jumps across the inversion of the horizontal velocity components, $z_{i}$ is the inversion height, and ${\hbox {d}z_i }/{\hbox {d}t}$ is the rate of change of the inversion height. $\Delta b$ is the jump across the inversion of the horizontally averaged buoyancy, and $B_{s}$ is the surface buoyancy flux, and *e* is the averaged TKE, ${\Phi }_{i}$ is the flux due to upward radiation of energy from the top of the CBL and $\varepsilon$ is the dissipation rate of TKE. Terms 1 and 2 on the right-hand side represent surface shear production, and term 3 represents shear production at the top of the boundary layer, across the entrainment zone.
